# The role of moral reasoning & personality in explaining lyrical preferences

**DOI:** 10.1371/journal.pone.0228057

**Published:** 2020-01-24

**Authors:** Kyle J. Messick, Blanca E. Aranda

**Affiliations:** Brain, Belief, & Behaviour Lab, Coventry University, Coventry, England; University of Vermont, UNITED STATES

## Abstract

Previous research has supported that personality traits can act to a precursor to media preferences. Due to the ongoing association between morality and media preferences in public and political discourse (e.g., blaming immoral behaviours on media preferences), this research sought to expand the knowledge about factors that contribute to media preferences by investigating if moral reasoning styles explain some of the variance that was not already explained by personality traits. A specific form of media preferences were chosen – lyrical preferences in metal music – as claims between metal lyrical themes and behaviour have been ongoing since the 1980s, despite a lack of empirical evidence to support these claims. A lyrical preferences scale was developed, and utilizing this scale, it was found that different types of metal fans exhibit different moral reasoning styles dependent on their metal sub-genre identification. Further, it was found that moral reasoning styles explain a portion of the variance in lyrical preferences that weren’t already explained by personality traits. In particular, lyrical preferences were often thematically consistent with moral reasoning content and personality traits, such as that individuals that preferred lyrics about celebrating metal culture and unity had higher levels of the group loyalty moral reasoning domain alongside being higher in extraversion. The implications of moral reasoning styles and personality traits as being precursors to media preferences are discussed.

## Introduction

There is an ongoing debate in popular culture about the relationship between various forms of violent media, negative behaviour, and immorality. This article focuses on the example of music, from which generalities are often made between socially taboo lyrics (e.g., content that includes sexuality, violence, drugs, suicide, etc.) and actual behaviour. The associations between lyrics and immoral behaviour often don’t hold up under scrutiny, and studies rarely account for lyrical preferences and their precursors. Currently there is evidence of many precursors to media preferences, including emotional vulnerability/content relatability [1], personality factors [2, 3] and an individual’s pre-existing ideology [4]. This study builds on previous studies of media preferences by using a pre-established precursor–personality traits–as a basis to look at an additional potential precursor: moral reasoning styles. Understanding how moral reasoning relates to lyrical preferences can help with understanding the relationship between morality and media preferences.

Previous literature suggests that individuals embrace lyrics which are associated with certain stable characteristics of that person, including ideology and personality traits, which are explained later in the present article. This article will also critically review popular media arguments which claim that certain controversial lyrics ‘corrupt’ music listeners, rather than music listeners seeking out those lyrics because they are consistent with their pre-existing moral reasoning styles. Following this, the main focus of this article is about the role of moral reasoning and personality traits in predicting lyrical preferences.

### The relationship between metal music and behaviour

Despite metal fans being consistently stereotyped as immoral [5,6, 7, 8] and empirical data showing that they are no less moral than their non-metal counterparts in regards to aspects like violent imagery processing [9], the subject of metal fans' morality persists in being a topic of public interest due to the imagery and lyrical content associated with metal music—often interpreted by non-metal fans as morally and socially taboo. Metal music, along with other genres such as rap music, have been especially targeted by critics who claim that the lyrics associated with those genres of music lead to violence, adolescent sexuality, and misogyny. These fear tactics have been used to garner public support for the regulation of metal music [10], as well as for jazz [11], rock, and rap music [12]. Similar claims have been used to censor other forms of consumptive entertainment, including literature [13, 14], film [15], and video games; however, the focus of this research is on metal music. Metal music is an intense form of counter-cultural music that is heavily associated with sensation-seeking and arousal [16], which is probably why it is associated with deviance as well. Heavy metal music is frequently associated with drug use, suicide, and other negative behaviours, but there is no obvious causal relationship, as this musical preference is often indicative of pre-existing emotional vulnerability [17]. Even for those metal fans that indulge in drug use as a coping mechanism early in life, their metal cultural identities work as a protective factor from negative outcomes [18]; furthermore, there are often gender and cultural differences in whether or not that association is accurate [19].

The association between metal lyrics and negative behaviour in popular media largely began in 1985 when an American committee, known as the Parents Music Resource Centre (PMRC), led a movement that put strict restrictions on the purchasing of music with crude lyrical themes. The PMRC specifically targeted a number of rock and metal bands, including Judas Priest, Mötley Crüe, AC/DC, Twisted Sister, W.A.S.P., Mercyful Fate, Black Sabbath, Def Leppard, and Venom. These artists were targeted for their lyrical subject matter, which dealt with sex, violence, vulgar language, the occult, and drug use. The PMRC reduced the accessibility of metal albums with violent, drug-related or sexual themes by labeling albums with Parental Advisory stickers if they included any of that content. The legacy of politicians and media outlets citing metal music as dangerous or stating that it could lead to negative behaviour continues today, such as when a school shooting occurs and the shooter happens to be a fan of metal music with violent lyrics. The media assume a causal link, or at least promote a causal link in their attention-grabbing headlines between metal music lyrics including bands such as Judas Priest, Slayer, and Ozzy Osbourne with violent acts or suicide, even though empirical investigation has found that these violent offenders actively search for lyrics that enforce their own pre-existing ideas about revolutionary violence [4]. It is not the case that the shooters commit acts because of the lyrics, but rather, they seek out lyrics that reinforce views that they already have. Further, although those associations are frequently debunked, there is little in-depth in the literature about metal lyrics and their relationship with moral reasoning. This is particularly true when focusing on extreme metal music subgenres, which have large and global fanbases, but that frequently have much more socially taboo lyrics than what popular artists like Judas Priest and Ozzy Osbourne incorporate. For instance, brutal death metal and slam are subgenres of death metal that are largely distinguished by lyrics about the torture, forceful penetration, and ritual murder of people, often exclusively women.

Black metal can have largely anti-Christian lyrics that embrace Satan and Satanism as a positive and guiding force. Depressive suicidal black metal (DSBM) focuses exclusively on extremely negative emotions, including suicidal ideation, which is also found to an extent in doom metal. Often, more extreme lyrics are meant to be counter-cultural by embracing taboo topics and themes, and even without that intentionality, they’re likely to be perceived that way within a broader, non-metal cultural context [20]. Music fans in general frequently listen to music because it relieves stress, and that effect is all the stronger when the individual chooses the music that they listen to due to the fundamentally important role of personal preferences [21]. This may also extend to other forms of consumptive media, as it has been found that violent media can often be used as a coping mechanism to deal with pre-existing stressors [21]. Additionally, in the specific case of metal music, it has been found to serve as a buffer for death anxiety [22]. Further, even though metal music is stereotypically associated with negative emotions and behaviours, metal music fans can have lower levels of depression and anxiety than the general population [23], and negative behaviours from members of music cultures are often associated with the behaviour of peers rather than the music itself [24].

Metal music, and particularly extreme subgenres like death metal, are frequently regarded as violent media comparable to horror movies or violent video games. As previously noted, violent media is frequently associated with negative or violent behaviour [25, 26], but those studies are frequently called into question due to methodological issues or publication bias [27]. Some studies even conclude that there is an inverse relationship between violent media and actual violence, as is the case with video games [28, 29]. There is limited research investigating the relationship between lyrical preferences and violence, but a recent study found that those who prefer violent lyrics are no less repulsed by violent imagery than those who do not prefer those lyrics, meaning that fictitious violence (at least in lyrical form) does not desensitize people to real violence [29]. Similar effects have been investigated in regard to other types of lyrics. For instance, it has been claimed that prosocial lyrics can lead to prosocial behaviour [30, 31, 32], although that finding cannot always be replicated [33]. The effect of lyrics on behaviour is not unique to prosocial lyrics, as studies have found that aggressive lyrics can lead to more aggression, romantic lyrics can lead to increased compliance in a courtship request [34] and misogyny can be decreased using pro-equality lyrics [35]. However, all of these studies use experimental settings with predetermined music with lyrics as stimuli, which is far different from the experience of listening to music in real life. People typically seek out music based on their own preferences, rather than being given lyrical content that was chosen for them by a researcher and within a lab setting. They also fail to take *context* into consideration, since being provided violent lyrics in an experimental setting is likely different from listening to preferred music with violent lyrics in a setting intended to fulfil a specific function (e.g., motivation) from aggressive music, such as at the gym. Subsequently, a distinction should be made between being experimentally primed with lyrics versus an ecologically valid setting where you choose your music.

It has been found that metal music serves as stress-relief for fans of metal music, which is contrary to claims that it induces violent behaviour [36]. These positive effects are not limited to lyrical content either, as the musical structure of metal music also has alleviating effects for some users [37], and these qualities extend to other styles of music too, as long as they are genres that the listener prefers [38]. The relationship between metal music and violence is especially weak when accounting for mediating factors. For example, personality factors can predispose people to liking violent media, making any relationship with violence reflective of the person’s personality, rather than their choice of media entertainment [2, 3]. Violent lyrics have been associated with hostile feelings [39], but this study failed to control for lyrical preferences. It is likely that violent lyrics have a different influence on an individual who prefers violent lyrics than on a person who is unwillingly bombarded with lyrics that they find to be immoral, disgusting, uncomfortable, and/or disturbing. Generally, people who listen to music with violent lyrics choose to do so, rather than being forced to do so, causing a lack of generalizability in past research findings. The present study makes the distinction between different types of lyrical preferences to account for one of the main methodological problems with the literature [39].

### Explaining the lyrical scapegoat: Moral panic theory

Before exploring moral reasoning as a precursor to lyrical preferences, it’s important to understand why metal lyrics, and other forms of violent media such as video games or other genres of music, are blamed for immoral actions despite empirical evidence of the contrary. One of the leading explanations for why video games and music are blamed for violence following tragic events is moral panic theory which:

…suggests a panic or overreaction to forms of deviance or wrong doing believed to be threats to the moral order. Moral panics are usually framed by the media and led by community leaders or groups intent on changing laws or practices…Moral panics gather converts because they touch on people’s fears and because they also use specific events or problems as symbols of what may feel to represent ‘all that is wrong with the nation’. [40].

Similarly, Stan Cohen developed a processual model of moral panic that is one of most widely utilized moral panic models. He described moral panic this way:

Societies appear to be subject, every now and then, to periods of moral panic. A condition, episode, person or group of persons emerges to become defined as a threat to societal values and interests; its nature is presented in a stylized and stereotypical fashion by the mass media; the moral barricades are manned by editors, bishops, politicians and other right-thinking people; socially accredited experts pronounce their diagnoses and solutions; ways of coping are evolved or (more often) resorted to; the condition then disappears, submerges or deteriorates and becomes more visible. Sometimes the object of the panic is quite novel and at other times it is something which has been in existence long enough, but suddenly appears in the limelight. Sometimes the panic passes over and is forgotten, except in folk-lore and collective memory; at other times it has more serious and long-lasting repercussions and might produce such changes as those in legal and social policy or even in the way the society conceives itself. [41].

Cohen’s model has three stages. In the first stage claims are exaggerated and distorted; in the second a terrible outcome is predicted if action is not taken (e.g. banning metal music, as the Parent Music Resource Center attempted in the 1980s), and in the third stage problems are symbolised as in associating the term ‘rocker’ with threat [42].

Another widely utilized moral panic model is that of Erich Goode and Nachman Ben-Yehuda in 1994. Theirs is an attributional model that challenged the assumption that social problems could be defined, measured, explained, and ameliorated [43]. They described five characteristics of moral panics, including: (1) concern, where there is a heightened level of concern about certain groups or categories, (2) hostility, where one can observe an increase in hostility towards the ‘deviants’ of ‘respectful society’, (3) consensus, where a consensus about the reality and seriousness of a threat can be found, (4) disproportionality, where public concern is in excess of what ‘should’ be, and (5) volatility, where the panic is temporary and fleeting and though it might reoccur, the panic is not long lasting.

While there are differences in both models, they both maintain a focus away from the validity of claims and their justifications, and instead focus on the dynamics of the social changes that occur when a threat is perceived. Moral panic theory has been applied in the context of many social problems, such as in response to street crime, drug and alcohol consumption, immigration, child abuse (including pedophilia), and media technologies [44]. It serves a polarizing role in its use of placing blame on particular explanations for immoral behaviour, such was the case in the ‘sexual dangers’ of jazz and swing music in the 1930s & 40s [10], and causes people to defend or firmly stand with explanations that put the blame for violence on video games and musical lyrics, be it rap music, or as is applicable in the current context, metal lyrics. Often the end result or ‘solution’ is a change in regulations, law, or the way in which a law is enforced. In the metal context, as mentioned earlier, it resulted in the development of the Parent Music Resource Center (PMRC), who further sought governmental intervention to regulate metal lyrics [10]. However, since the perceived threats of metal lyrics (e.g., sexual promiscuity, violence) were largely only perceived threats and not actual threats, the laws and regulations that were created in response to them were more of a formality than reflective of a societal change in attitudes, to reinforce the values of a society that could not condone vulgar lyrical content [42].

It should be noted that moral panic theory is not without its critics, who claim that these theories are outdated as they do not have an updated view of mass media, their operationalization is often vague, they don’t include a focus on the promoters and supporters of moral panics, and they ignore insights from recent theories about moral regulation of risk, among others [44, 45]. However, they offer a widely accepted explanation as to why violent media, including metal lyrics, are often the target of societal blame when tragedy occurs. This contextualizes popular associations between lyrics and behaviour, but it doesn’t explain where lyrical preferences stem from.

### Moral reasoning and media consumption

Moral Foundations Theory (MFT) is a social intuitionist perspective which states that human moral reasoning originates from innate, modular foundations. It assumes that morality is a trait-like disposition that guides behaviour, attitudes, and communication through content-specific evaluative procedures that act as motivating principles. It was created by a group of social and cultural psychologists to understand how morality varies across different cultures. MFT has been shown to work across different cultures [46], but this is the first study to apply it to metal culture. MFT states that the usage of different moral foundations is based on interactions between biology, cultural socialization, and individual experiences [47]. Moral foundations have previously been used to explore the relationships between moralities and political ideology, personality, stereotyping, attitudes, emotion, cultural differences, intergroup relations, moral cognition, and more [48]. Moral foundations have been associated with a number of behaviours and attitudes that distinguish different types of people, such as actions towards climate change [49], religion [50], political alignment [51], or vegetarianism [52]. Due to MFT’s wide and successful application across multiple cultures, it was selected to explore the relationship between moral reasoning and lyrical preferences in metal culture, and will also be applied to investigating differences in types of metal fans.

The five moral domains that encompass MFT are intended to be innate, global psychological systems from which unique moralities can be drawn. Each of the foundations provides a point within a dichotomous spectrum between polar opposites, with the moral end being the higher scoring end. For example, someone with a higher score on care/harm morally reasons towards being more caring, and less harming towards others. The first foundation, care/harm, reflects the ability to empathize with the struggles of others. The second foundation, fairness/cheating, is about reciprocity and concerns about equality. It is from this moral foundation that ideas of justice and proportionality are derived. The third domain is loyalty/betrayal, which reflects how humans live in groups, how they can be patriotic towards that group, and are willing to self-sacrifice for their group. Authority/subversion is the fourth moral domain, which is about leadership and followship, respect for traditions, and how cultures can have a hierarchical social structure. Lastly, sanctity/degradation is the last moral domain, which is heavily influenced by the psychology of disgust and contamination. This final moral domain ties into religious beliefs, and living a less carnal, nobler life, where the body is seen as a holy place that should not be desecrated.

MFT has previously been applied to explore the relationship between moral foundations and other forms of media preferences. For instance, the salience of multiple moral foundations has been found to predict moral reasoning while playing video games. Further, it has been suggested that playing violent video games could change moral reasoning toward real-life violence, but more research is needed before making that conclusion [53]. Gamers often claim that there is a separation between how real violence and video game violence is processed, and unjustifiable violence in video games generates guilt responses similar to real violent scenarios, indicating that video games can trigger moral responses in users [54]. Further, feelings of guilt and negative affect in violent video games are reduced by framing the game as ‘just a game’ or by justifying the violence through familiarity with the game or through the narrative [55], thus how violent media is contextualized and interpreted seems to play an important role in understanding its relationship with moral behaviours. Outside of video games, moral foundations have been used to understand how people enjoy violent content within television and movies, using MFT as a way to divide subcultural responses to violent media [56, 57]. In the case of violence in metal music, the descriptions are often so violent that they’re claimed to be a parody or cartoonishly unrealistic, thus separating them from real violent events, but it hasn’t yet been explored how moral reasoning relates to these forms of lyrical preferences and if these lyrical preferences reflect an individual’s moral reasoning processes, or if, like in the case of video games, these violent depictions are processed similarly to real acts of violence.

## The current study

Moral reasoning styles explain many characteristics of people, including attitudes [58] and preferences. The main hypothesis in this study is that lyrical preferences stem, at least in part, from moral reasoning styles. Evidence has already been found that moral reasoning styles act as a precursor for preferences, as is the case of ideological preferences [59], but little has been connected thus far in regards to more common, everyday preferences, such as lyrical preferences. In the context of this article, the focus is on lyrical preferences among heavy metal music fans. Heavy metal music culture is a conglomerate of distinct music cultures that differ in sound, aesthetic, and cultural norms [60]. Each subgenre of metal has specific types of lyrics, which makes it a possible way to account for some of the major cultural differences across metal music fans. For instance, heavy metal and power metal are genres that frequently have lyrical themes about metal fans unifying, embracing metal culture, having pride, and fighting honourably. Black metal frequently has more melancholic themes such as darkness, Satanism, and is frequently about struggling in cold climates. Death metal appropriately and stereotypically deals with the topic of death, often detailing murders in gruesome, horrifying, or sexually depraved ways. Doom and progressive metal frequently deal with lyrical themes that are about struggle and inner emotional turmoil.

It is expected that lyrical preferences are firstly shaped by personality traits, and secondarily by moral reasoning styles. This will be tested in the following way: 1) by developing a lyrical preferences scale that categorizes similar lyrical preferences into factors that can be used to compare different types of music listeners, 2) by examining if there are moral reasoning differences between types of metal fans, 3) testing the relationship between moral foundations and lyrical preferences, and 4) investigating the additional role of moral reasoning alongside personality types in explaining metal lyrical preferences.

## Method

### Participants

The Coventry University Board of Ethics approved this study (reference number: P63157). A total of 1,237 people participated in the study, but only the 341 completed surveys were included in the analyses. There weren’t any restrictions for participation, other than that participants should be at least 18 years of age, although the recruitment specifically targeted fans of metal music. There was not a significant relationship found between the country of origin of participants and either moral foundations or the lyrical factors, so participants were not excluded from analysis based on country of origin. The rationale behind this decision was that metal music culture, including cultural norms and rituals, can be consistent across political lines [37], and that all participants were part of the same online metal music community. Of the 341 participants, 261 (76.5%) identified as male, 73 (21.4%) as female, 4 (1.2%) as transgender, and 3 (.9%) as other. Participants were primarily white (N = 286, 83.9%), but other ethnic groups were also present (Black/African descent, 1.5%; Asian descent, 2.1%; Latino, 3.5%; Other, 9%). The ‘other’ category had a variety of answers, most of which were about being biracial or mixed. The sample represented a wide spectrum of ages, with the oldest participant being 60 (Mean age = 31, SD = 9.5). The invitations to participate in the study were directed specifically at metal fans, mostly in sub-genre specific metal chat groups. The discussion groups were largely focused on exchanging metal music, experiences, comparing bands, attending metal music festivals, or exchanging metal-themed memes. Most of the sample were born in the United States (46.3%), followed by the United Kingdom (11.7%). Countries represented were diverse, including Australia, Iran, India, Korea, Mexico, Norway, Turkey, South Africa, Zambia, and more. The broad spectrum of regions in the sample gives credence to the global extent to which metal culture exists and is interconnected because general, non-region specific metal groups were exclusively targeted for recruitment. Most of the sample reported their marital status as single, never married (58%; married or living as, 37%; widowed, .6%; separated or divorced, 4.4%). When asked for religious affiliation, the largest number of metal fans checked the box for “none” (31.4%) which reflects a stereotypical counter-cultural disinterest by many metal fans for religion and religious categorization. An additional 28.7% considered themselves atheist, and 12.3% agnostic. A minority of participants identified themselves as Satanic (2.6%) or Pagan (5.3%). The rest identified themselves as Buddhist (.3%), Christian (14.4%), Islamic (.6%), Judaism (.9%), or other (3.5%). The number of Christians in the sample might be somewhat disproportionately over-represented because a few Christian metal groups were specifically advertised to. Many that fell into the “other” category called themselves a hybrid of affiliations, such as agnostic atheist, agnostic pagan, atheistic Satanist, and Pagan/Deist/Satanist.

The educational level of the participants varied greatly, from 2.3% not having finished high school, and 3.5% having completed a PhD. Most participants were either high school graduates (17.6%), attended some college (26.1%) or had obtained a Bachelor’s degree (27%) or Master’s degree (15%). Other categories included Associate degree (4.7%) and other post-college degrees (3.8%). Economic status varied greatly as well, with 35.8% making less than $16,000 a year, 3.5% making more than $100,000 a year, and the rest of the sample falling somewhere in-between. These demographic characteristics appear representative of members of metal music culture, particularly for those in extreme subgenres of metal, as they are consistent with the metal demographics found by Guibert and Guibert which showed that fans of metal music are represented across all education and income levels, and primarily come from a white ethnic background, are mostly male, and are overwhelmingly non-religious [61].

### Materials and measures

The survey included general demographic items as well as measures for lyrical preferences (e.g., To what extent do you enjoy lyrics about love and romance?), the Big Five Inventory (BFI; [62]), and the 20-item short version of the Moral Foundations Questionnaire (MFQ), which had participants determine the extent to which they agreed with a series of moral statements, (e.g., One of the worst things a person could do is hurt a defenceless animal) [63]. The reliabilities for the BFI were satisfactory (agreeableness α = .76, conscientiousness α = .76, extraversion α = .82, neuroticism α = .84, openness α = .72). The scores on the MFQ items were added, so each participant received a possible score out of 24 for each moral foundation (6-point Likert scale, 4 items per foundation). The reliabilities for each of the five Moral Foundations subscales can be found in Table 1. Each of the subscales consisted of four items.

**Table 1:** Cronbach’s Alpha levels for the moral foundations subscales.

Moral Foundations Scale	Cronbach’s Alpha
Care / Harm	.66
Fairness / Cheating	.59
Loyalty / Betrayal	.60
Authority / Subversion	.66
Sanctity / Degradation	.71

#### Measuring metal lyrical preferences

A list of common lyrical content of songs for different music genres was compiled, which was used to develop a list of 35 common lyrical topics. In the current study, we used a 5-point Likert scale (1 = I greatly dislike lyrics like this, 2 = I don’t like lyrics about this, 3 = I’m indifferent to lyrics about this, 4 = I like lyrics about this, 5 = I greatly enjoy lyrics about this) to ask about lyrical preferences.

Following principle component analysis using the Varimax Rotation technique with Kaiser normalization and the analysis of a scree plot we found five factors. The component cut-off value for exclusion was set at .30. The only lyrical theme that did not load into any factor was a preference for lyrics of religious themes, thus that lyrical preference was excluded from further analyses. The individual items and their respective factor loadings within each factor can be seen on Table 2, and the breakdown of these factors their reliabilities can be found on Table 3. In instances where items loaded into more than one factor, the factor was placed within the most conceptually similar factor.

**Table 2:** Factor items & factor loading for lyrical preferences.

**Factor 1: Vulgar/immoral lyrics**	**1**	**2**	**3**	**4**	**5**
- Sexual intercourse	.67	.36			
- Horror	.58				
- Extreme sexual acts	.81				
- Lyrics that are over-the-top and completely ridiculous	.41	.40			
- The dismemberment and/or forceful penetration of women	.74				
- Bodily functions, such as peeing, pooping, or about a woman’s menstrual cycle	.70				
- Gory lyrics about themes such as mutilation and murder	.78				
- Anything that is taboo	.63				
- Gang-related activities	.48	.35			
- Anti-Christian or Satanic lyrics	.47	-.32			
**Factor 2: Lyrics that embrace metal culture and fun**					
- Unity		.61			
- Metal and being a part of metal		.60			
- Money and getting rich		.54			
- Lyrics about empowerment and conquering obstacles		.46	.44		
- Honor and pride		.47	.50		
- Humorous		.61			
- Partying/drinking		.60			
- Cheesy lyrics		.50			
**Factor 3: Lyrics about the human experience**					
- Deep, emotional themes			.81		
- Tranquility		.35	.59		
- Love and romance		.43	.44		
- Heavy topics about things like depression and emotional turmoil			.76		
- Hardships, struggle			.73		
- Peace			.57		
**Factor 4: Lyrics embracing history, mythology, and nature**					
- War / battle				.76	
- Mythologies, such as Norse or Egyptian gods				.69	
- Fantasy lyrics, such as about dragons and knights				.60	
- Landscapes and seasons, such as mountains and winter			.41	.53	
- Historical time frames				.73	
**Factor 5: Lyrics about science and science fiction**					
- Sci-fi lyrics					.86
- Zombies or some other monsters	.41				.60
- Scientific exploration and/or outer space					.72
- Aliens					.80
- Lovecraftian themes and stories					.53

**Table 3:** Five factors representing lyrical subject preferences.

Lyrical Factor	Number of items	Cronbach’s Alpha
Vulgar / Immoral	10	.84
Embracing Metal Culture/Fun	8	.76
Human Experience	6	.80
History/Myths/Nature	5	.77
Science and Science Fiction	5	.79

### Procedure

An online questionnaire was distributed through social networking sites, but through specifically targeted groups that had a focus on metal music and its subgenres. Some of these groups required the researcher to answer metal culture historical questions in order to gain entry to the group and post in it (e.g., Which band referred to themselves as “hydro-grind?” Answer: Cephalic Carnage) or to agree to certain terms (e.g., You will agree not to post about Cannibal Corpse or other bands that everyone already knows about). Many of these groups had an extensive list of rules prior to posting, so the researcher only posted a link to the online questionnaire in cases where it did not violate the rules of the group. Some groups did not have rules or conditions for posting, so there was a huge difference in the formality and regulation of each group. When participants clicked on the survey link, they read over a study information page and then an informed consent page. Participation in the study was voluntary, and participants were allowed to quit the study at any time without consequence. None of the participants received compensation for their participation, and there weren’t any anticipated risks or benefits, although 46.6% (N = 149) of participants said that they enjoyed or really enjoyed taking the survey. The survey began with general demographic items, including about the type of metal fan that the participant most identities as, then participants filled out the lyrical preference and moral foundations measures.

## Results

Analyses were conducted to gain insight into the moral reasoning of metal fans. I aimed to establish: 1) the relationship between the type of metal fan and moral foundations;, and 2) the extent to which moral foundations and personality traits explain lyrical preferences. The first relationship was explored using an ANOVA, and the second through a correlation to establish the relationships, and then through hierarchical regressions to clarify the direction of the relationship.

### The relationship between type of metal fan and moral foundations

An exploratory one-way ANOVA was run to determine if there were differences in moral reasoning between types of metal fans. Nine types of metal fans were explicitly compared (black metal, death metal, doom metal, grind/power violence, heavy metal, power metal, thrash, folk metal, progressive metal), with 314 participants for this analysis. See Table 4 for a full list of descriptive statistics. Homogeneity of variance was not violated for any of the five moral foundation factors. It was found that there were significant differences on the dimensions of authority/subversion, F(9,331) = 1.91, p = .05, η2 = .04, and sanctity/degradation, F(9,331) = 2.94, p = .002, η2 = .08, but not for care/harm, fairness, or group loyalty foundations. Metal fans had similar scores for care/harm (M = 19.13, SD = 3.65), fairness (M = 19.28, SD = 3.17), and group loyalty moral priorities (M = 12.62, SD = 4.09), regardless of their preferred metal subgenre. Bonferroni multiple comparisons tests were run to explore those differences, but likely due to the smaller sample sizes of fans outside of black metal fans (N = 83) and death metal fans (N = 78), there was only a significant difference detected between those two groups on sanctity/degradation. It was found that black metal fans had significantly lower scores on sanctity/degradation moral foundation (M = 11.22, SD = 4.30) than death metal fans (M = 13.95, SD = 4.57), p = .007. This is consistent with the ideology and lyrical content of black metal, which is frequently anti-religious.

**Table 4:** Mean scores and standard deviations of nine types of metal fans across five moral domains.

	Black	Death	Doom	Grind	Heavy	Power	Thrash	Folk	Prog
**Care**	18.57	19.63	20.05	19.07	18.36	19.41	18.35	19.46	19.08
	(3.96)	(3.24)	(3.19)	(3.97)	(4.49)	(3.57)	(4.02)	(3.07)	(2.47)
**Fairness**	18.58	19.95	20.05	19.14	18.82	19.32	18.96	19.08	19.08
	(3.45)	(2.84)	(2.14)	(2.82)	(3.87)	(3.98)	(2.99)	(2.78)	(3.58)
**Group** **Loyalty**	12.05	13.05	12.59	13.14	13.75	11.91	13.30	12.62	10.75
	(4.14)	(4.05)	(3.92)	(5.08)	(4.42)	(4.22)	(2.72)	(4.44)	(3.60)
**Authority**	11.45	13.42	12.29	11.79	13.39	12.36	11.52	13.31	10.03
	(4.43)	(4.44)	(3.91)	(5.55)	(4.49)	(5.19)	(4.12)	(4.77)	(3.94)
**Sanctity**	11.22	13.95	12.49	12.43	13.79	13.82	11.65	13.62	9.58
	(4.30)	(4.57)	(4.56)	(4.36)	(4.52)	(4.71)	(4.99)	(3.88)	(5.28)

### Moral foundations as explaining the variance in lyrical preferences

The five types of lyrical preferences were normally distributed across participants. Pearson correlations were run between the five lyrical factors, the five moral domains, and the five personality traits. The results of the correlations can be found in Table 5.

**Table 5:** Correlations between lyrical preferences with moral reasoning and personality factor.

Variable	Factor	Lyrical Factor				
		Vulgar/ Immoral	Embracing Metal Culture & Fun	Human Experience	History/ Mythology/ Nature	Science & Science Fiction
Moral reasoning	Care/harm	-0.09	.15**	.32***	0.06	.15**
	Fairness	-0.1	0.1	.22***	0.02	0.06
	Group loyalty	-0.04	.25***	0.04	.14**	-0.04
	Authority/Subv.	-0.1	.24***	0.02	0.04	-0.01
	Sanctity/Degrad.	-.23***	.18***	.13*	-0.01	-0.06
Personality style	Agreeableness	-.12*	.13*	.19**	0.07	0.02
	Conscientiousness	-0.05	0.07	0.03	-0.07	0.02
	Extraversion	0.063	.15**	-0.08	-0.04	0.01
	Neuroticism	0.09	0.06	.16**	.11*	0.1
	Openness	0.04	0.08	.12*	.13*	.18***

* p < .05

** p < .01

*** p < .001

Preferring lyrics about human experience correlated positively with the care/harm, fairness, and sanctity/degradation moral dimensions. Lyrics about embracing history, mythology, and nature only correlated with the group loyalty moral domain. A preference for science and & science fiction lyrics only correlated with the care/harm moral domain. Enjoying lyrics about embracing metal culture and having fun were positively correlated with the care/harm, group loyalty, authority/subversion, and purity/sanctity moral domains. The only lyrical preference with a significant negative relationship was that between vulgar/immoral lyrics and the sanctity/degradation moral domain.

There were also significant associations found between lyrical preferences and personality traits. Agreeableness was negatively correlated with a preference for vulgar/immoral lyrics. Enjoying lyrics about embracing metal culture & fun was positively associated with agreeableness and extraversion. Preferring lyrics about the human experience was positively related to agreeableness, neuroticism, and openness to experience. Enjoying lyrics about history, mythology, and nature was positively associated with neuroticism and openness. Enjoying lyrics about science and science fiction was only associated with higher levels of openness to experience.

It was predicted that moral reasoning styles and personality types might predict and explain some of the variance in lyrical preferences, thus a series of five hierarchical multiple regression analyses were performed using the five moral foundations to predict variability in lyrical preferences. A two stage hierarchical multiple regression was conducted with a lyrical preference factor serving as the dependent variable in each analysis. Personality variables were used as predictors in stage one, as personality types had previously been established as playing a role in media preferences [2,3]. Moral reasoning styles were entered at stage two. A summary of the results can be found in Table 6.

**Table 6:** Summary of hierarchical regression analyses for variables predicting lyrical prefrences .

**Regression 1: Preference for vulgar/immoral lyrics**				** **	** **	** **
**Variable**	**B**	***t***	**sr**^**2**^	***R***	***R***^**2**^	***ΔR***^**2**^
Step 1				0.171	0.029	0.029
Agreeableness	-0.106	-1.900	0.010			
Conscientiousness	-0.025	-0.423	0.001			
Extraversion	0.103	1.754	0.009			
Neuroticism	0.088	1.754	0.009			
Openness	0.031	0.559	0.001			
Step 2				0.290	0.084	0.055
Care	-0.034	-0.497	0.001			
Fairness	-0.03	-0.461	0.001			
Group Loyalty	0.068	0.982	0.003			
Authority	0.035	0.456	0.001			
Purity	-0.259***	3.848	0.041			
**Regression 2: Preference for lyrics that embrace metal culture & fun**						** **
**Variable**	**B**	***t***	**sr**^**2**^	***R***	***R***^**2**^	***ΔR***^**2**^
Step 1				0.249	0.062	0.062
Agreeableness	.137*	2.494	0.017			
Conscientiousness	.067	1.162	0.004			
Extraversion	.179**	3.090	0.027			
Neuroticism	.185**	3.040	0.026			
Openness	.025	0.454	0.001			
Step 2				0.385	0.148	0.086
Care	.073	1.107	0.003			
Fairness	.037	1.107	0.001			
Group Loyalty	.155*	2.313	0.014			
Authority	.151*	2.029	0.011			
Purity	.008	0.116	0.000			
**Regression 3: Preference for lyrics about the human experience**					** **	** **
**Variable**	**B**	***t***	**sr**^**2**^	***R***	***R***^**2**^	***ΔR***^**2**^
Step 1				0.320	0.103	0.103
Agreeableness	.228***	4.246	0.048			
Conscientiousness	.076	1.334	0.005			
Extraversion	-.064	-1.135	0.003			
Neuroticism	.230***	3.876	0.040			
Openness	.113*	2.116	0.012			
Step 2				0.407	0.166	0.063
Care	.193**	2.956	0.022			
Fairness	.057	0.917	0.002			
Group Loyalty	.057	0.858	0.002			
Authority	-.051	-0.694	0.001			
Purity	.094	1.456	0.005			
**Regression 4: Preference for lyrics embracing history, mythology, & nature**						** **
**Variable**	**B**	***t***	**sr**^**2**^	***R***	***R***^**2**^	***ΔR***^**2**^
Step 1				.206	0.043	0.043
Agreeableness	.098	1.772	0.009			
Conscientiousness	-.062	-1.061	0.003			
Extraversion	-.026	-0.448	0.001			
Neuroticism	.107	1.741	0.009			
Openness	.142*	2.571	0.019			
Step 2				.288	0.083	0.040
Care	.017	0.254	0.000			
Fairness	-.037	-0.570	0.001			
Group Loyalty	.239***	3.425	0.033			
Authority	-.022	-0.281	0.000			
Purity	-.084	-1.251	0.004			
**Regression 5: Preference for lyrics about science & science fiction**						
**Variable**	**B**	***t***	**sr**^**2**^	***R***	***R***^**2**^	***ΔR***^**2**^
Step 1				.214	0.046	0.046
Agreeableness	.027	0.481	0.001			
Conscientiousness	.034	0.573	0.001			
Extraversion	.012	0.210	0.000			
Neuroticism	.132*	2.146	0.013			
Openness	.175**	3.172	0.029			
Step 2				.259	0.067	0.021
Care	.150*	2.173	0.013			
Fairness	-.014	-0.221	0.000			
Group Loyalty	-.037	-0.531	0.001			
Authority	.105	1.357	0.005			
Purity	-.103	-1.522	0.007			

* p < .05

** p < .01

*** p < .001

The hierarchical multiple regression revealed that at Stage one, personality traits did not contribute significantly to the preference for vulgar/immoral lyrics regression model, F (5,335) = 2.01, p = .08). However, introducing the moral reasoning styles explained an additional 5.6% of variation in preferring vulgar/immoral lyrics and the change in R^2^ was significant, F (10,330) = 60.10, p = .001. For preferring lyrics that embraced metal culture and fun, Stage one of the analysis revealed that 4.8% of the variance was explained by personality traits, F (5,335) = 4.43, p = .001. The addition of moral reasoning styles accounted for 12.2% of the variance in enjoying lyrics about metal culture and fun, F (10, 330) = 5.73, p < .001. For lyrics about the human experience, 8.9% of the variance was accounted for by personality traits, F (5, 335) = 7.65, p < .001, whereas 14.1% was accounted for when combined with moral reasoning styles, (10, 330) = 6.56, p < .001. At Stage one for preferring lyrics about history, mythology, and nature, personality traits explained 2.8% of the variance, F (5,335) = 2.98, p = .01, and with Stage two, the combination with moral reasoning styles explained 5.5%, F (10, 330) = 2.98, p = .001. Lastly, 3.2% of the variance in enjoying lyrics about science & science fiction was explained by personality traits, F (5, 335) = 3.22, p = .008, and with moral reasoning styles 6.7% of the variance was accounted for, F (10, 330) = 2.38, p = .01. Overall, the personality trait of Openness was the one that played a significant role in predicting more types of lyrical preference.

## Discussion

This study examined the association between lyrical preferences, moral reasoning domains, and personality traits. We first developed a lyrical preferences scale that allowed for comparisons between members of the metal music community. We then identified differences about moral reasoning based on the type of metal preference (e.g., black metal fans had the lowest scores on the sanctity/degradation moral foundation), and further analyses revealed a rather nuanced understanding about how lyrical preferences are related to moral foundations.

Correlations showed that enjoying lyrics about depression, hardships, love, and emotional turmoil (i.e. human experience) are related to having higher scores on the care/harm and fairness moral domains, which suggests that those people who prefer these lyrics might find the struggles of others related to their own hardships, and might be more empathetic to those struggles. Individuals who showed a preference for vulgar lyrics, such as misogyny, violence, and Satanism in their lyrical content, have significantly lower scores on the sanctity/degradation moral domain compared to other metal fans. One possible explanation is that these metal music fans have a different understanding of sanctity (e.g., thinking of the body as a temple), which is materialised in how these music fans with vulgar/immoral lyrical preferences have higher levels of extreme and unusual piercings and body modifications than metal fans with other lyrical preferences (something we recorded in this study, but was not a focus of the analysis). It is also possible that those who prefer more disgusting lyrics are approaching the idea of disgust in a fundamentally different way than most other music and metal music fans. Adding further cause for concern, the sanctity/degradation subscale has recently been questioned for how inconsistently it applies to people who are not religious [64], which is primarily the case for metal fans in the current sample, as most participants identified themselves as atheistic, agnostic, or ‘none.’ This moral domain has been further criticized, as it has been claimed that ‘purity’ is a descriptive label, and does not reflect a form of moral processing [65]. Future research could explore the relationship between the sanctity of the body and the self for metal fans, particularly for those that prefer immoral/vulgar lyrics.

A preference for lyrics about embracing metal culture & fun, which include themes like unification and loyalty, was associated with higher scores on the moral domains for authority/subversion, group loyalty, care/harm, and sanctity/degradation. It is expected that lyrics about loyalty would be associated with higher levels of loyalty as a moral foundation, which would extend into the authority moral domain, since this shows an appreciation of leadership and followship, as well as a respect for traditions. These lyrics celebrate metal culture, which might extend to a more formal view of metal culture, via respect for traditions, loyalty to the metal community, increased levels of caring, and more common views on purity compared to those who prefer vulgar/immoral lyrics and more extreme metal subgenres. This is a direction that future research could further explore. Lyrics about honour and pride include sentiments that fit well into a worldview that has structure and respect for that structure.

A preference for lyrics about history, mythology, and nature, which include lyrics about war, patriotism, and martyrdom, was positively associated with the group loyalty moral foundation. This association is reasonable, as lyrics about war can emphasize themes such as loyalty to a nation, regime, or historical entity, so individuals who enjoy these are likely seek out lyrics about loyalty as this is a moral principle important to them.

Having a preference for lyrics about science and science fiction only had a relationship with the care/harm moral domain. These are likely lyrics that are more morally neutral, which might explain the lack of an association with other moral domains. The association between science and science fiction lyrics and the moral foundation that focuses on empathy and struggle is not an intuitive one, so future research could explore this relationship by encompassing preferences for this sort of content in other forms of media, such as science fiction films.

Personality traits also had a relationship with lyrical preferences, including vulgar/immoral lyrical preferences negatively relating to agreeableness. People that enjoyed lyrics about embracing metal culture and fun had personality traits that reflected being more extraverted and agreeable, so this lyrical style that embraces activities with groups and friends is consistent with being outgoing, sympathetic, and trusting of others. Preferring lyrics about the human experience was positively related to being more agreeable, neurotic, and open to experience, showing that choosing to listen to emotional lyrics about human experiences is often consistent with having a personality associated with feelings like anxiety, depression, and loneliness. In other words, there was consistency between the lyrical subject matter and the emotional experiences of the person. Similarly, choosing to listen to lyrics about history, nature, and mythology was associated with higher levels of neuroticism and openness to experience. Lastly, enjoying lyrics about science & science fiction were only related to higher levels of openness to experience. Openness to experience is likely associated with lyrics about history and science fiction because these are lyrical topics that describe experiences that might be far from the user’s everyday experiences, indicating a preference towards lyrics that have interesting and often unfamiliar narratives that satiates their openness and desire for new experiences.

These relationships were given further clarification by investigating the extent to which moral reasoning and personality traits explained lyrical preferences. It was found that moral foundations do explain a unique and significant portion of the variance in lyrical preferences that was not already accounted for by personality traits. For example, preferring lyrics about human struggle was related to the moral foundation for care in addition to higher levels of neuroticism and agreeableness. In this instance, an individual that has higher levels of care and empathy involved in how they morally reason might also prefer lyrics about human emotions and struggles that similarly use their ability to be empathetic, since some of their personality traits (neuroticism) suggest that a tendency towards emotional instability.

In relation to the insights from moral panic theory, this study clarifies the direction between moral reasoning and lyrical preferences adding support to the hypothesis that lyrical preference reflect pre-existing moral foundations and personality traits, rather than lyrics leading to a ‘corruption’ of morality. This is consistent with previous findings showing that those who perform acts of violence seek out lyrics that justify their pre-existing beliefs about violence [4].

This study has also brought insights to the metal studies literature, showing that adhering to a metal subgenre has significant implication. For example, black metal fans had lower levels in the sanctity/purity moral domain, which is consistent with the frequently anti-religious message of black metal culture [66].

A few limitations should be noted. First, the moral foundations subscales had slightly lower internal reliabilities than anticipated. It is worth nothing that recently, Moral Foundations Theory (MFT) has been criticized for how difficult it is to replicate across cultures [67]. These limitations might extend into metal culture, in which case MFT might not be the ideal way to explore the moral reasoning of people within metal music culture. Further, the lyrical factor scale developed here comes with its own limitations. It is not expected that the factors presented would be universal across every music culture since lyrical themes and meanings vary across cultural contexts. Although the different types of lyrics could be used in the study of other music cultures, it is likely that the factor structure of the scale would be different. For instance, gang-related activities fall under the vulgar/immoral factor for metal fans, but they might not fall into the same factor in hip hop music, since lyrical topics like Satanism (also part of the vulgar/immoral factor) probably don’t appeal to the same types of hip hop fans as gang-related lyrics. A future study could investigate the degree to which lyrics are interpreted as entertaining, relational, or as a statement of endorsement towards a certain perspective (e.g., pro-violence). This meaning-making approach to lyrics would allow for understanding the role and functions of lyrical preferences, with some functional possibilities including escapism, coping, or feeling like someone else understands a user’s emotional turmoil. Other future studies could evaluate how behaviour directly relates to moral reasoning and media preferences, since it is known that moral foundations don’t always correlate with moral behaviours [68]. Whereas it is the case that violent media, such as video games [28, 29], can reduce violent behaviours, a future study could see if listening to music with violent lyrical themes serves a similar function.

This study, which explored the relationship between lyrical preferences, moral preferences and personality traits will hopefully add more nuance to the discussion of types of music and their association with moral and immoral behaviours. We suggest that lyrical preferences might originate in pre-existing characteristics, including moral reasoning styles and personality traits. This evidence can be added to that of other precursors for media preferences, including emotional vulnerability/relatability [1], personality factors [2, 3], and pre-existing ideology [4]. Given this, it is hoped that future associations between media (not limited to metal music), and behaviour will take into account these psychological factors, since there is now evidence that moral orientation is as similarly fundamental as personality traits in predicting media preferences.
